# Optimal Concordant Tests

**DOI:** 10.3390/app14114536

**Published:** 2024-05-25

**Authors:** Zhongxue Chen

**Affiliations:** College of Health Solutions, Arizona State University, 425 N. 5th Street, #137, Phoenix, AZ 85004, USA;

**Keywords:** chi-square test, constrained likelihood ratio test, gamma distribution, meta-analysis, uniformly most powerful test

## Abstract

In meta-analyses, unlike model-based methods, such as fixed- or random-effect models, the p-value combining methods are distribution-free and robust. How to appropriately and powerfully combine p-values obtained from various sources remains an important but challenging topic in statistical inference. For cases where all or a majority of the individual alternative hypotheses have the same but unknown direction, concordant tests based on one-sided p-values can substantially improve the detecting power. However, there exists no test that is uniformly most powerful; therefore, figuring out how to choose a robust and powerful test to combine one-sided p-values for a given data set is desirable. In this paper, we propose and study a class of gamma distribution-based concordant tests. Those concordant tests are optimal under specific conditions. An asymptotically optimal concordant test is also studied. The excellent performances of the proposed tests were demonstrated through a numeric simulation study and real data example.

## 1. Introduction

Due to recent technical developments, larger volume data, including genome-wide genomic data, can be generated rapidly and at a low cost. As a consequence, powerful statistical approaches, including p-value combination tests, are highly desirable for analyzing these data sets. For instance, meta-analyses, when applied to genome-wide association studies (GWASs), have discovered many associated genetic variants that could not be identified from a single GWAS. With the development and application of advanced powerful p-value combination tests, we expect that many more casual genetic variants will be identified through existing data.

In meta-analyses, p-value combination methods are important alternatives to model-based techniques [1,2], especially when the fixed- or random-effect models have a lack of fit [3]. In the literature, many p-value combination methods, although first proposed a long time ago, are still widely used nowadays. These methods include popular ones: Fisher test [4], Pearson test [5], minimal p-test [6], z-test [7], and chi-square test [8,9]. Recently, we studied a class of p-value combination tests based on gamma distribution, which include those popular tests as special cases [10]. However, as noticed by Birnbaum, there exists no uniformly most powerful (UMP) p-value combination test for all conditions [11]. Therefore, robust methods that have good power under many conditions are desirable [12].

Under some conditions, we may know or reasonably assume that all or most of the true effects under the individual alternative hypotheses have the same but unknown direction. In this case, the concordant tests which use one-sided p-values have been shown to be more powerful than those methods based on two-sided p-values, such as the chi-square test [1]. For instance, in GWASs, studies with the same phenotype are usually conducted among several different subpopulations, from which the effect of a causal genetic variant may have the same direction but various sizes. Then, if the commonly used fixed- and random-effect meta-analyses are applied to combine the results from individual studies, they may lose the power to detect the associated genetic variants. To improve the detecting power, in this paper, we study a class of concordant tests based on gamma distribution, and then an optimal concordant test based on constrained likelihood ratio test (CLRT) is developed.

The rest of the manuscript is organized as follows. In Section 2, we first introduce the Pearson’s concordant test, which was studied by Owen [1] (Section 2.1). In Section 2.2, we describe our proposed generalized concordant tests based on gamma distribution and study their properties as UMP tests under some conditions. The asymptotically UMP concordant test based on CLRT is proposed and studied in Section 2.3. In Section 3, we compare the performances of the proposed concordant tests with some existing methods through a simulation study. In addition, an example of a real data application is illustrated to demonstrate the desired performance of the proposed tests. This paper concludes in Section 4 with a discussion and conclusions.

## 2. Methods

Suppose we have n independent studies. For each study, i(i=1,⋯,n), we can perform a two-sample test for testing the null hypothesis, $H_{i0}:\mu_{i}=0$, against the alternative hypothesis, $H_{i1}:\mu_{i}\neq 0$, where $\mu_{i}$ is the mean difference between two populations. The corresponding p-value is denoted as $P_{i}(i=1,\cdots ,n)$. Throughout this paper, we assume $P_{i}\sim \text{U}(0,1)$ under $H_{i0}$, where U(0, 1) stands for the uniform distribution between 0 and 1. In a meta-analysis, we consider testing the global null hypothesis, $H_{0}=\cap H_{i0}$, vs. the global alternative hypothesis, $H_{1}=\cup H_{i1}$, using the n observed p-values.

For a concordant test, we assume, under the global alternative, that the majority of the non-zero mean differences have the same but unknown direction (positive or negative): $\mu_{i}\geq 0$ and at least one strictly greater than holds, or $\mu_{i}\leq 0$ and at least one strictly less than holds (i=1,⋯,n). Although, under this setting, a two-sided p-value can still be calculated from each data set and then used in p-value combination, in this paper, we focus on the concordant tests which use one-sided p-values, $P_{i}$, obtained from the directional alternatives $\mu_{i}>0$ or $\mu_{i}<0$ for all i values (i=1,⋯,n). In general, either the right- or left-sided p-values can be used, as they will produce the same result. Without loss of generality, we use the right-sided individual p-values hereafter, unless otherwise specified.

### 2.1. Pearson’s Concordant Test

Owen [1] revisited and studied the following Pearson’s concordant test, whose test statistic is defined as follows:

(1) $$Q^{C}=\text{max}\left(-2\sum_{i=1}^{n}\text{ln}\left(P_{i}\right),-2\sum_{i=1}^{n}\text{ln}\left(1-P_{i}\right)\right),$$

where $P_{i}(i=1,\cdots ,n)$ represents the one-sided p-values obtained from the same directional alternatives. The two components, $-2\sum_{i=1}^{n}\text{ln}\left(P_{i}\right)$ and $-2\sum_{i=1}^{n}\text{ln}\left(1-P_{i}\right)$, in $Q^{C}$ are obtained through the popular Fisher test for combining independent p-values [4]. Therefore, under the global null hypothesis, each component is the random variable, $\chi_{2n}^{2}$, which has a chi-square distribution with degrees of freedom equal to 2n. Furthermore, although $-2\sum_{i=1}^{n}\text{ln}\left(P_{i}\right)$ and $-2\sum_{i=1}^{n}\text{ln}\left(1-P_{i}\right)$ are not independent, the p-value of the Pearson’s concordant test defined in (1) can be easily approximated using the upper bound, $2P\left[\chi_{2n}^{2}>q^{\text{C}}\right]$, where $q^{C}$ is the observed test statistic. In addition, the approximation is very accurate when the true p-value is small [1].

### 2.2. Concordant Tests Based on Gamma Distribution

The above Pearson’s concordant test can be extended to concordant tests based on gamma distribution. For this purpose, we first study a class of combination tests based on gamma distribution [10].

For given shape parameter α and scale parameter β, a p-value combination test statistic based on the gamma distribution can be constructed as follows:

(2) $$T_{G(\alpha ,\beta )}\left(P_{1},P_{2},\cdots ,P_{n}\right)=\sum_{i=1}^{n}F_{G(\alpha ,\beta )}^{-1}\left(1-P_{i}\right),$$

where $F_{G(\alpha ,\beta )}^{-1}(y)$ is the inverse function of the cumulative distribution function (CDF), $F_{G(\alpha ,\beta )}(x)$, of the random variable Gamma(α,β), whose probability density function (PDF) is $f_{G(\alpha ,\beta )}(x)=\beta^{\alpha }x^{\alpha -1}\text{exp}(-\beta x)/\Gamma (\alpha )$ for x>0, where the gamma function $\Gamma (z)=\int_{0}^{\infty }x^{z-1}e^{-x}dx$. The right-sided p-value of the test defined in (2) can be defined as follows:

(3) $$P=P(\text{Gamma}(n\alpha ,\beta )>t)=1-F_{G(n\alpha ,\beta )}(t)=S_{G(n\alpha ,\beta )}(t),$$

where Gamma(nα,β) is the random variable having gamma distribution with the shape parameter, nα, and the scale parameter, β; and $F_{G(n\alpha ,\beta )}$ and $S_{G(n\alpha ,\beta )}$ are the CDF and the survival function of Gamma(nα,β).

It can be shown that, due to the property of gamma distribution, parameter β has no effect on the test defined in (2) and (3) [10]. Therefore, for simplicity, we use β=1 in the above test and denote $T_{G(\alpha ,1)}$ as $T_{G(\alpha )}$ hereafter. Furthermore, we can prove the following properties for the above class of gamma distribution-based tests [10]: (i) $T_{G(0)}≜\underset{\alpha \to 0+}{\text{lim}}T_{G(\alpha )}$ is equivalent to the Tippett’s minimal-p test; (ii) $T_{G(0.5)}$ is the same as the chi-square test with degrees of freedom equal to n; (iii) $T_{G(1)}$ is equavalent to Fisher test; and (iv) $T_{G(\infty )}≜\underset{\alpha \to \infty }{\text{lim}}T_{G(\alpha )}$ is the same as the z-test. Therefore, the class of gamma distribution-based tests, $T_{G(\alpha )}$, includes many popular tests as special cases. This observation motivates us to generalize the Pearson’s concordant test.

We propose the gamma distribution-based concordant test with the following test statistic:

(4) $$T_{G(\alpha )}^{C}=\text{max}\left(T_{G(\alpha )}^{L},T_{G(\alpha )}^{R}\right),$$

where $T_{G(\alpha )}^{L}=T_{G(\alpha )}\left(P_{1},P_{2},\cdots ,P_{n}\right)=\sum_{i=1}^{n}F_{G(\alpha )}^{-1}\left(1-P_{i}\right)$ and $T_{G(\alpha )}^{R}=T_{G(\alpha )}\left(1-P_{1},1-P_{2},\cdots ,1-P_{n}\right)=\sum_{i=1}^{n}F_{G(\alpha )}^{-1}\left(P_{i}\right)$ are the gamma distribution-based tests using the left- and right-sided p-values, respectively.

It is easy to see that when $\alpha =1,T_{G(\alpha )}^{C}$ is equivalent to the Pearson’s concordant test $Q^{C}$. Therefore, $T_{G(\alpha )}^{C}$ is a generalization of the Pearson’s concordant test. We show that the p-value for the proposed concordant test, $T_{G(\alpha )}^{C}$, can also be estimated in a similar way as $Q^{C}:P\left[T_{G(\alpha )}^{C}>A\right]\approx 2P\left[T_{G(\alpha )}^{L}>A\right]$. Now, we study the properties of $T_{G(\alpha )}^{C}$. First we need the following definitions and lemmas [13].

**Definition 1**. *A function* f *on* $R^{n}$ *is nondecreasing if it is nondecreasing in each of its* n *arguments when the other* n−1 *values are held fixed*.

**Definition 2**. *Let* $X_{1},\cdots ,X_{n}$ *be random variables with a joint distribution. These random variables are associated if the covariance* Cov(f(X),g(X))≥0 *holds for all nondecreasing functions,* f *and* g, *for which the covariance is defined*.

**Lemma 1**. *(Theorem 2.1 of Esary, Proschan, and Walkup 1967* [13]) *Independent random variables are associated*.

**Lemma 2**. *(Theorem 5.1 of Esary, Proschan, and Walkup 1967* [13]) *Let* $X_{1},\cdots ,X_{n}$ *be associated random variables*, $X=\left(X_{1},\cdots ,X_{n}\right),S_{i}=f_{i}(X),i=1,\cdots ,k$, *then* $P\left[S_{1}\leq s_{1},\cdots ,S_{k}\leq s_{k}\right]\geq \prod_{i=1}^{k}\left[S_{i}\leq s_{i}\right]$ *and* $P\left[S_{1}>s_{1},\cdots ,S_{k}>s_{k}\right]\geq \prod_{i=1}^{k}\left[S_{i}>s_{i}\right]$ *for all* $s_{1},s_{2},\cdots ,s_{k}$.

**Lemma 3**. *For integer* n≥1*, let* $X=\left(X_{1},\cdots ,X_{n}\right)\in (0,1{)}^{n}$ *have independent components. Set* $T_{G(\alpha )}^{L}=T_{G(\alpha )}\left(X_{1},\cdots ,X_{n}\right)$*, and* $T_{G(\alpha )}^{R}=T_{G(\alpha )}\left(1-X_{1},\cdots ,1-X_{n}\right)$. *Then, for any* $A_{L}>0$*, and* $A_{R}>0,P\left[T_{G(\alpha )}^{L}>A_{L},T_{G(\alpha )}^{R}>A_{R}\right]\leq P\left[T_{G(\alpha )}^{L}>A_{L}\right]P\left[T_{G(\alpha )}^{R}>A_{R}\right]$.

**Proof**. Denote $f(X)=T_{G(\alpha )}^{L}=-\sum_{i=1}^{n}F_{G(\alpha )}^{-1}\left(1-P_{i}\right)$ and $g(X)=T_{G(\alpha )}^{R}=\sum_{i=1}^{n}F_{G(\alpha )}^{-1}\left(P_{i}\right)$. It is easy to see that both f and g are nondecreasing functions of X. And the components of X are independent and hence associated. Therefore, we have

$$P\left[T_{G(\alpha )}^{L}>A_{L},T_{G(\alpha )}^{R}>A_{R}\right]=P\left[-f(X)>A_{L},g(X)>A_{R}\right]=P[f(X)<\left.-A_{L},g(X)>A_{R}\right]=P\left[g(X)>A_{R}\right]-P\left[f(X)>-A_{L},g(X)>A_{R}\right]\leq P\left[g(X)>A_{R}\right]-P\left[f(X)>-A_{L}\right]P\left[g(X)>A_{R}\right]=P\left[g(X)>A_{R}\right]P[f(X)<\left.-A_{L}\right]=P\left[T_{G(\alpha )}^{R}>A_{R}\right]P\left[T_{G(\alpha )}^{L}>A_{L}\right].$$

□

The concordant test, $T_{G(\alpha )}^{C}$, defined in (4) has the following property.

**Theorem 1**. *Let* $X=\left(X_{1},\cdots ,X_{n}\right)\in (0,1{)}^{n}$ *be a random vector with independent components, and denote* $T_{G(\alpha )}^{L}=T_{G(\alpha )}\left(X_{1},\cdots ,X_{n}\right),T_{G(\alpha )}^{R}=T_{G(\alpha )}\left(1-X_{1},\cdots ,1-X_{n}\right)$*, and* $T_{G(\alpha )}^{C}=max\left(T_{G(\alpha )}^{L},T_{G(\alpha )}^{R}\right)$. *For any* A∈R, *let* $P_{L}=P\left[T_{G(\alpha )}^{L}>A\right],P_{R}=P\left[T_{G(\alpha )}^{R}>A\right]$*, and* $P_{C}=P\left[T_{G(\alpha )}^{C}>A\right]$. *Then* $P_{L}+P_{R}-P_{L}P_{R}\leq P_{C}\leq P_{L}+P_{R}$

**Proof**. From Lemmas 2 and 3, we have $P_{C}=P\left[T_{G(\alpha )}^{C}>A\right]=P\left[T_{G(\alpha )}^{L}>A\;\text{or}\;T_{G\left(\alpha_{0}\right)}^{R}>A\right]=P\left[T_{G(\alpha )}^{L}>A\right]+P\left[T_{G(\alpha )}^{R}>A\right]-P\left[T_{G(\alpha )}^{L}>A,T_{G(\alpha )}^{R}>A\right]\geq P\left[T_{G(\alpha )}^{L}>A\right]+P\left[T_{G(\alpha )}^{R}>A\right]-P\left[T_{G(\alpha )}^{L}>A\right]P\left[T_{G(\alpha )}^{R}>A\right]=P_{L}+P_{R}-P_{L}P_{R}$. On the other hand, from Bonferroni inequality, we have $P_{C}\leq P\left[T_{G(\alpha )}^{L}>A\right]+P\left[T_{G(\alpha )}^{R}>A\right]=P_{L}+P_{R}$. □

Under the condition specified in Theorem 1 (i.e., the global null hypothesis), it is easy to see that $T_{G(\alpha )}^{L}$ and $T_{G(\alpha )}^{R}$ have the same distribution; hence, $P\left[T_{G(\alpha )}^{L}>A\right]=P\left[T_{G(\alpha )}^{R}>A\right]$.

From Theorem 1, we have the following result.

**Corollary 1**. *Suppose* $X\sim U(0,1{)}^{n}$*, for* A>0*, let* $p_{A}=P\left[T_{G\left(\alpha \right)}^{L}>A\right]$, *then* $2p_{A}-{p_{A}}^{2}\leq P\left[T_{G\left(\alpha \right)}^{C}>A\right]\leq 2p_{A}$.

From Corollary 1, the p-value of the concordant test, $T_{G(\alpha )}^{C}$, defined in (4), $P\left[T_{G(\alpha )}^{C}>A\right]$, can be easily approximated by twice of the p-value from $T_{G(\alpha )}^{L}$, i.e., $P\left[T_{G(\alpha )}^{C}>A\right]\approx 2P\left[T_{G(\alpha )}^{L}>A\right]$. The approximation is very accurate if the true p-value (e.g., the p-value from the sampling distribution under the alternative) is small, as ${p_{A}}^{2}$ is even much smaller under this situation.

In the following subsection, we study an optimal concordant test when the optimal value for α is unknown and needs to be estimated from the data.

### 2.3. Optimal Concordant Test

For a given set of one-sided p-values, the class of concordant tests, $T_{G(\alpha )}^{C}$, proposed in the previous section, Section 2.2, with different α values can be applied. However, in order to control type I error, the α value must be chosen before we see the data. But usually, the optimal value is unknown, and therefore how to choose an optimal value for α is critical in practice. We may choose a set of α values to apply the tests $T_{G(\alpha )}^{C}$ to the data and then use the smallest p-value among them. There are two limitations associated with this procedure. First, we need to adjust for multiple comparisons, which may result in power loss. Second, the final result will depend on the chosen α values; it might be difficult to determine those values. To circumvent these difficulties, we propose to estimate the optimal α value from the data, and therefore an asymptotically optimal concordant test can be constructed accordingly.

In this subsection, we make the following assumption: $P_{1},P_{2},\cdots ,P_{n}$ are independently and identically distributed (*iid*) with the following density function for parameters α>0 and c<1:

(5) $$f_{\alpha ,c}(p)=(1-c{)}^{\alpha }\text{exp}\left[cF_{G\left(\alpha \right)}^{-1}\left(1-p\right)\right]\;\text{for}\;p\in (0,1)\text{.}$$

For the above densify function, $f_{\alpha ,c}(p)$, it is not difficult to show that when $c=0,f_{\alpha ,0}(p)=1(0<p<1)$. Therefore, $f_{\alpha ,0}(p)$ corresponds to the global null hypothesis. In addition, we have the following property [10].

**Proposition 1**. *Suppose that* $P_{1},P_{2},\cdots ,P_{n}$ *are iid with the common density function defined in* (5) *with parameters* α>0 *and* c<1. *If both* α *and* c *are known, then the gamma distribution-based test*, $T_{G(\alpha )}$*, is uniformly most powerful (UMP)*.

When both parameters α and c in (5) are unknown, they can be estimated via the constrained maximum likelihood estimation (CMLE), from which the following constrained likelihood ratio test (CLRT) can be defined:

(6) $$T_{\text{CLRT}}\left(P_{1},\cdots ,P_{n}\right)=2{\overset{ˆ}{\alpha }}_{\text{CLRT}}n\text{ln}\left(1-{\overset{ˆ}{c}}_{\text{CLRT}}\right)+2\left({\overset{ˆ}{c}}_{\text{CLRT}}\right)\sum_{i=1}^{n}F_{G\left({\overset{ˆ}{\alpha }}_{\text{CLRT}}\right)}^{-1}\left(1-P_{i}\right),$$

where ${\overset{ˆ}{\alpha }}_{\text{CLRT}}$ and ${\overset{ˆ}{c}}_{\text{CLRT}}$ are the CMLEs for parameters α and c, respectively, through maximizing the log-likelihood function $l(\alpha ,c)=n\alpha \text{ln}(1-c)+c\sum_{i=1}^{n}F_{G(\alpha )}^{-1}\left(1-P_{i}\right)$ with the constrains c<1 and α>0.

For the above CLRT-based test, $T_{\text{CLRT}}$, we have the following result [10,14].

**Proposition 2**. *The asymptotic distribution of the test* $T_{\text{CLRT}}$ *is a mixture of chi-square distributions*, $\sum_{i=0}^{2}w_{i}\chi_{i}^{2},\text{where}\;\chi_{i}^{2}$ *is the chi-square distribution with* $df=i;\chi_{0}^{2}$ *is the random variable with probability 1 of being 0; and the weights* $w_{0},w_{1}$*, and* $w_{2}$ *are determined by the null and the alternative hypothesis*.

The above asymptotic result may not be directly applicable to estimate the p-value for this test. First, the number of p-values, n, is usually small, and the asymptotic result may not provide approximate solutions. Second and more seriously, the above weights, $w_{i}’s$, are difficult to obtain. Hence, here we use a simple resampling method to approximate the null distribution and to estimate the p-value of $T_{\text{CLRT}}$. More specifically, for a given sample size n, we randomly sample n independent null p-values from the uniform distribution U(0,1) and then calculate the test statistic using (6). Repeat this process for B times (e.g., $B=10^{5}$) to get the empirical null distribution of the test statistic, which will be used to approximate the p-value of $T_{\text{CLRT}}:p=\#\left[T_{i}\geq T_{\text{CLRT}}\left(p_{1},\cdots ,p_{n}\right)\right]/B$, where $T_{i}(i=1,\cdots ,B)$ is the test statistic from the $i^{\text{th}}$ resampling, and $T_{\text{CLRT}}\left(p_{1},\cdots ,p_{n}\right)$ is the observed test statistic from the data.

For $T_{\text{CLRT}}$, we have the following result [10]:

**Proposition 3**. *Under the conditions specified in* (5), *the CLRT-based test*, $T_{\text{CLRT}}$*, is asymptotically UMP*.

We now propose an optimal concordant test. Denote $T_{\text{CLRT}}^{L}=T_{\text{CLRT}}\left(P_{1},P_{2},\cdots ,P_{n}\right)$, and $T_{\text{CLRT}}^{R}=T_{\text{CLRT}}\left(1-P_{1},1-P_{2},\cdots ,1-P_{n}\right)$, and then define the following concordant test statistic:

(7) $$T_{\text{CLRT}}^{C}=\text{max}\left(T_{LRT}^{L},T_{LRT}^{R}\right).$$

Note that the null distributions of $T_{LRT}^{L}$ and $T_{LRT}^{R}$ are identical. For the concordant test, $T_{\text{CLRT}}^{C}$, we have the following property.

**Theorem 2**. *Let* $X=\left(X_{1},\cdots ,X_{n}\right)\in (0,1{)}^{n}$ *be a random vector with independent components, and let* $T_{\text{CLRT}}^{L}=\underset{\alpha >0,c<1}{max}\left[n\alpha ln(1-c)+c\sum_{i=1}^{n}F_{G(\alpha )}^{-1}\left(1-X_{i}\right)\right],T_{\text{CLRT}}^{R}=\underset{\alpha >0,c<1}{max}\left[n\alpha ln(1-c)+c\sum_{i=1}^{n}F_{G(\alpha )}^{-1}\left(X_{i}\right)\right]$*, and* $T_{\text{CLRT}}^{C}=max\left(T_{\text{CLRT}}^{L},T_{\text{CLRT}}^{R}\right)$. *For any* A∈R, let $P_{L}=P\left[T_{\text{CLRT}}^{L}>A\right],P_{R}=P\left[T_{\text{CLRT}}^{R}>A\right],and\;P_{C}=P\left[T_{\text{CLRT}}^{C}>A\right]$. *Then*, $P_{L}+P_{R}-P_{L}P_{R}\leq P_{C}\leq P_{L}+P_{R}$.

**Proof**. First notice that $f(X)=-\underset{\alpha >0,c<1)}{\text{max}}\left[n\alpha \text{ln}(1-c)+c\sum_{i=1}^{n}F_{G(\alpha )}^{-1}\left(1-X_{i}\right)\right]$ and $g(X)=\underset{\alpha >0,c<1)}{\text{max}}\left[n\alpha \text{ln}(1-c)+c\sum_{i=1}^{n}F_{G(\alpha )}^{-1}\left(X_{i}\right)\right]$ are both nondecreasing functions of associated random variables, X. The rest of the proof is similar to that for Theorem 1. □

In addition, we have the following result for $T_{\text{CLRT}}^{C}$.

**Corollary 2**. *Suppose* $X\sim U(0,1{)}^{n}$*; for* A>0*, let* $p_{A}=P\left[T_{\text{CLRT}}^{L}>A\right]$*, and then* $2p_{A}-{p_{A}}^{2}\leq P\left[T_{\text{CLRT}}^{C}>A\right]\leq 2p_{A}$.

From Corollary 2, the p-value of the concordant test, $T_{\text{CLRT}}^{C}$, defined in (7) can be estimated by the upper bound, $2p_{A}$, where $p_{A}$ is the p-value from the CLRT-based test, $T_{\text{CLRT}}^{L}$, and can be estimated using the resampling method described previously. Again, when the true p-value is small, this approximation is very accurate.

## 3. Results

### 3.1. Numeric Results from Simulation Study

In this section, we assess the performance of the proposed tests through a simulation study. In the simulation, we compare the proposed optimal concordant test, $T_{\text{CLRT}}^{C}$, with gamma distribution-based concordant tests, $T_{G(0)}^{C},T_{G(1)}^{C}$ (i.e., the Owen’s Pearson concordant test), and $T_{G(\infty )}^{C}$, using one-sided p-values.

In the simulation study, we want to combine fifty (n=50) randomly simulated independent p-values using different methods. We assume that a number, m(m=10,20,40,50), out of these np-values are from the true individual alternative hypotheses, and the rest (n−m) are from the true individual null hypotheses. The n-m p-values from the true null hypotheses are randomly sampled from the uniform distribution with range 0 and 1. For a true individual alternative hypothesis, $H_{i1}(i=1,\cdots ,m)$, the p-value $p_{i}$ is obtained via a normal variable, $z_{i}\sim N\left(\mu_{i},1\right)$. To consider the situations where the true effects from different studies may have opposite directions (positive and negative), we randomly set k of the $m\;\mu_{i}\;s$ to have the same direction (positive or negative), and the rest of m−k has the other direction. A one-sided (right-sided) p-value for each true individual alternative hypothesis is obtained via the standard z-test, i.e., p=P[Z>z], where Z is the standard normal distribution, N(0,1), and z is the simulated number as described above.

For the hypotheses under true alternative, we consider three different scenarios for the effects of $\mu_{i}’s$. Scenario 1: We use $\left|\mu_{i}\right|=\mu v_{i}/\sum_{i=1}^{m}v_{i}$, where $v_{i}=10^{r_{i}},r_{i}\sim N(0.3,1)$, and μ=0.8,0.6,0.4,0.3 when there are 10, 20, 40, and 50 true individual alternatives, respectively. Scenario 2: We set $\left|\mu_{i}\right|=\mu v_{i}/\sum_{i=1}^{m}v_{i}$, where $v_{i}\sim U(1,100)$ and μ=1.2,1.0,0.6,0.5 when the number of true individual alternatives is m=10,20,40, and 50, respectively. Scenario 3: We consider $\left|\mu_{i}\right|=\mu /m,\mu =1.5,1.2,0.8$, and 0.6 when m=10,20,40, and 50, respectively. Note that, in the simulation, we consider the following: (i) The constants (e.g., μ, the parameters in the normal distribution for $r_{i}$ and the uniform distribution for $v_{i}$) are chosen in a way that the empirical powers are appreciable for comparison. (ii) The sum of the absolute effect sizes is equal to μ for all the three scenarios. (iii) The degree of heterogeneity of the effect sizes among the true individual alternatives decreases from Scenario 1 to Scenario 3 for each given m.

The empirical power values of the tests are estimated as the rejection proportions based on 1000 replicates, with 0.05 as the significance level. For the CLRT-based concordant test, $T_{\text{CLRT}}^{C}$, we use $B=10^{5}$ samples to estimate the p-values from the resampling method described in Section 2.3.

When all of the 50 p-values are from null hypotheses, the empirical power is the type 1 error rate. All methods considered here were able to control the type I error rate: their empirical powers under this situation were close to 0.05 when the significance level 0.05 was used.

Figures 1–3 plot the empirical power values for the concordant tests based on minimal $\text{p},T_{G(0)}^{C}$ (denoted as Min_CC), Fisher $T_{G(1)}^{C}$ (denoted as Fisher_CC, i.e., Pearson’s concordant test), z-test $T_{G(\infty )}^{C}$ (denoted as Z_CC), and the proposed concordant test, $T_{\text{CLRT}}^{C}$ (denoted as LRT_CC), when one-sided p-values under Scenarios 1 to 3, respectively, are used.

From the simulation study, we have the following observations. First, under Scenario 1 (Figure 1), where the effect sizes under alternative hypotheses are extremely heterogeneous, usually the minimal p-based concordant test (i.e., $T_{G(0)}^{C}$) performs better than the Pearson’s concordant test $\left(T_{G(1)}^{C}\right)$, which in turn outperforms the z-test-based concordant test $\left(T_{G(\infty )}^{C}\right)$. Second, under Scenarios 2 and 3, when the degrees of heterogeneity of the effect sizes among individual alternative hypotheses are less extreme than in Scenario 1, $T_{G(1)}^{C}$ and the $T_{G(\infty )}^{C}$ usually perform better or much better than $T_{G(0)}^{C}$ (Figures 2 and 3). Third, for the three concordant tests with fixed α values, namely $T_{G(0)}^{C},T_{G(1)}^{C}$, and $T_{G(\infty )}^{C}$, one may perform well under some conditions but do poorly under others. Fourth, under all conditions considered, the CLRT-based concordant test, $T_{\text{CLRT}}^{C}$, always has the best or the second-best power. This demonstrates that, as expected, $T_{\text{CLRT}}^{\text{C}}$ is a robust test, meaning that, under many conditions, it has reasonable detection power compared with other tests.

### 3.2. Real Data Application

In this section, we demonstrate the usefulness of the proposed tests by applying them to a real-world problem. A data set from a meta-analysis is used. In that meta-analysis, results from 12 randomized trials—each examined the effect of patient rehabilitation designed for geriatric patients versus usual care on improving functional outcome at 3–12-month follow-ups—were collected [15,16]. The 12 estimated odds ratios (ORs) from the individual studies are listed in Table 1.

The commonly used fixed-effect model for meta-analyses was inadequate for this data set, as the p-value from the Cochran’s test for homogeneity was 0.021. Hence, the random-effect model of the meta-analysis was applied, which estimated the overall OR as 1.36 with a 95% CI (1.08, 1.71) [16]. However, when we applied the goodness-of-fit test for the random-effect model, we obtained a p-value of 0.025 [3], which indicates a lack of fit of the random-effect model for this data set. We then applied the p-value combination methods to test whether there is an overall effect among these 12 independent trials.

To use the p-value combination methods, we need the individual p-values. For each trial, the corresponding p-value is calculated based on the reported 95% CI of the OR. If we use U and L to denote the upper and lower limits of the 95% CI, then the test statistic can be approximated as $z=\text{ln}(U\times L)/\sqrt{4\text{ln}(U/L)/3.92}$, which is asymptotically distributed as N(0,1) under the null hypothesis that there is no difference between the new treatment and the control. Since the sample sizes of these 12 trials were relatively large, ranging from 108 to 1388, their p-values can be reasonably estimated using the asymptotic null distribution by comparing the test statistic with N(0,1). For example, the right-sided p-value can be calculated as P[Z>z], where Z has a standard normal distribution, N(0,1), and z is the test statistic calculated as described above.

We first calculate the one-sided p-value for each trial and then use the values for the concordant tests. The p-values from the concordant tests $T_{G(0)}^{C},T_{G(1)}^{C},T_{G(\infty )}^{C}$, and $T_{\text{CLRT}}^{C}$ are 0.013, 0.00017, 0.00031, and 0.00030, respectively. For comparison, we also applied the gamma distribution-based tests to this data set, using two-sided p-values calculated from each individual trial. The resulting p-values are 0.013, 0.0068, 0.075, and 0.0047 from $T_{G(0)},T_{G(1)},T_{G(\infty )}$, and $T_{\text{CLRT}}$, respectively. As expected, under the situation that a majority of the trials have the same direction (10 out of the 12 estimated ORs were greater than 1), concordant tests are usually more powerful than the corresponding gamma distribution-based tests using two-sided p-values. Noticeably, the z-test-based concordant test, $T_{G(\infty )}^{C}$, obtained a much smaller p-value (0.00031) compared with the z-test using two-sided p-values (0.075). Among the concordant tests, the proposed test has the second smallest p-value (0.00030).

## 4. Discussion and Conclusions

In this paper, we studied a class of gamma distribution-based concordant tests, which include the Pearson’s concordant test as a special case. We also proposed and studied a CLRT-based concordant test, which is asymptotically optimal, robust, and powerful under many conditions. The advantages of the proposed tests were demonstrated based on numeric simulation study and real data application.

In addition to their use for meta-analyses, the proposed concordant tests have many other applications. In a two-way contingency table, if at least one of the two variables is ordinal, a trend test may be more powerful than the popular unidirectional Pearson’s chi-square test [17]. One challenge in trend tests is how to appropriately assign scores for each level of the ordinal variable; results can be quite different with different score assignments. Our proposed concordant test, $T_{\text{CLRT}}^{C}$, can circumvent this difficulty and provide another solution. Specifically, by using the asymptotically independent statistics with the same direction obtained by the Lancaster’s chi-square partition [18,19], we can obtain asymptotically independent one-sided p-values, to which $T_{\text{CLRT}}^{C}$ can be applied.

The proposed tests will also be very useful for detecting casual genetic variants associated with diseases when applied to GWASs. It offers an alternative but powerful tool to combine information from different (subpopulations) but related studies (similar phenotype). We would expect that many genetic variants will be identified in the near future due to the new approach. In the future, we will also study how to combine dependent or correlated p-values.

## Figures and Tables

**Figure 1.**
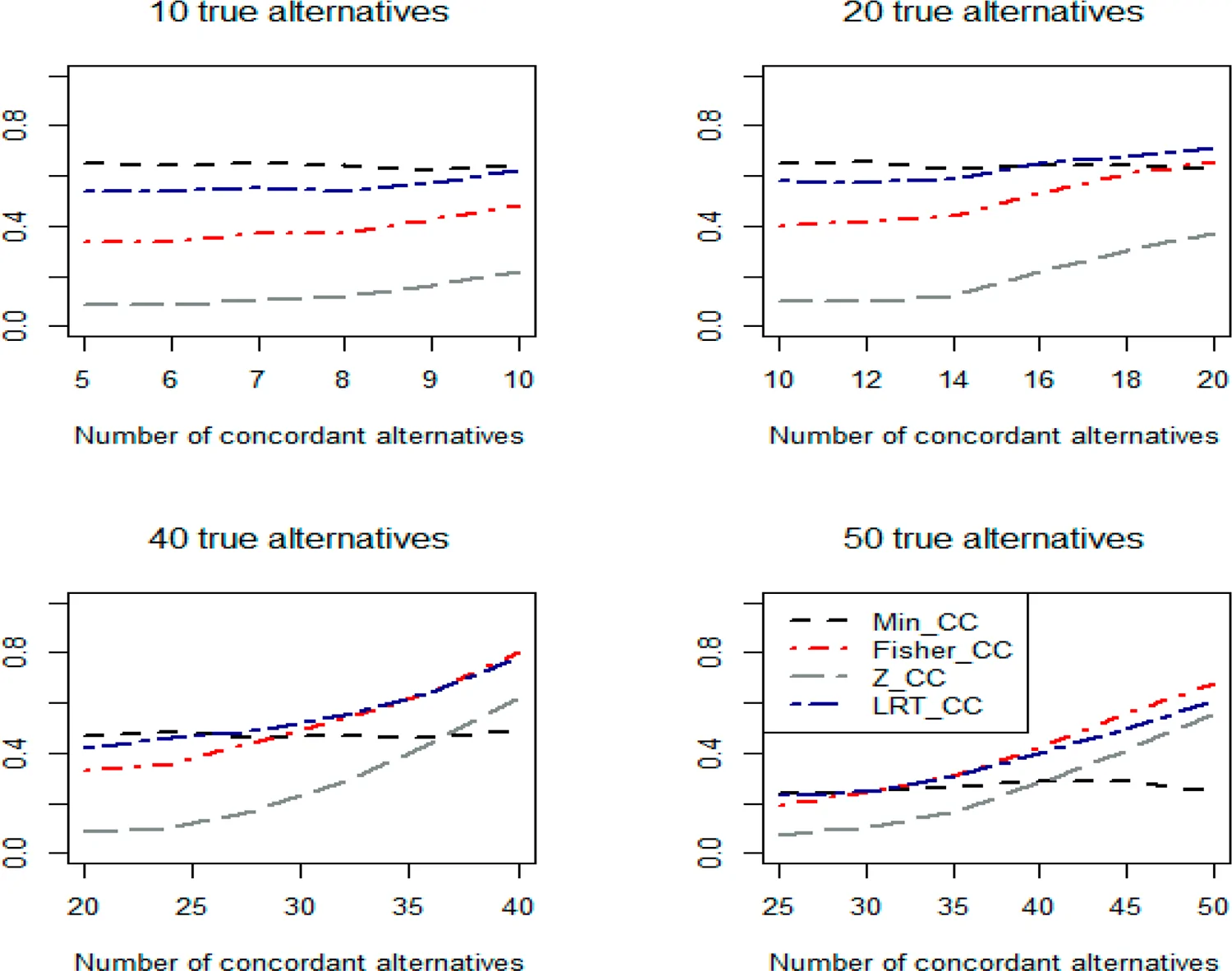
Empirical power values of the tests based on one-sided p-values under Scenario 1.

**Figure 2.**
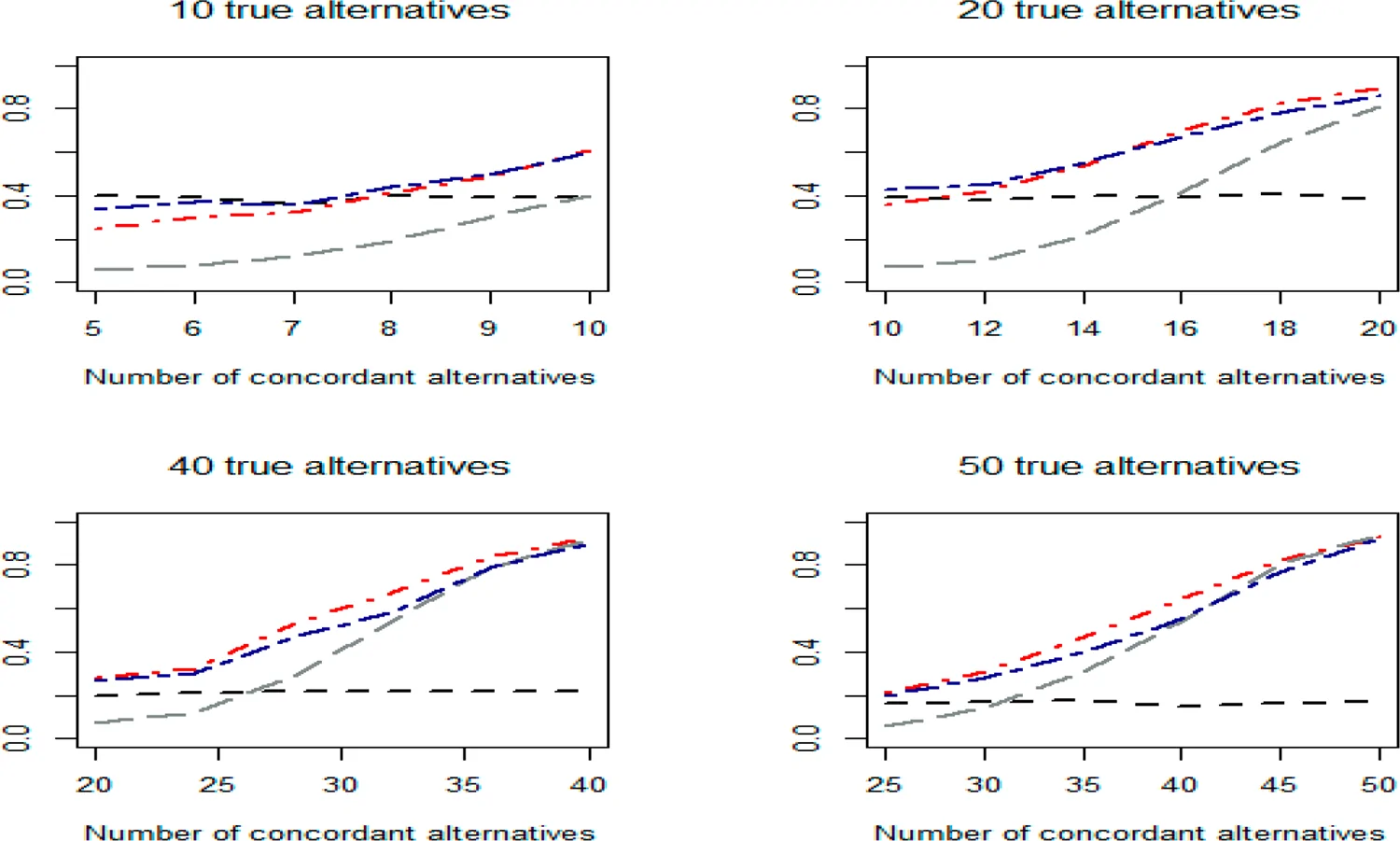
Empirical power values of the tests based on one-sided p-values under Scenario 2.

**Figure 3.**
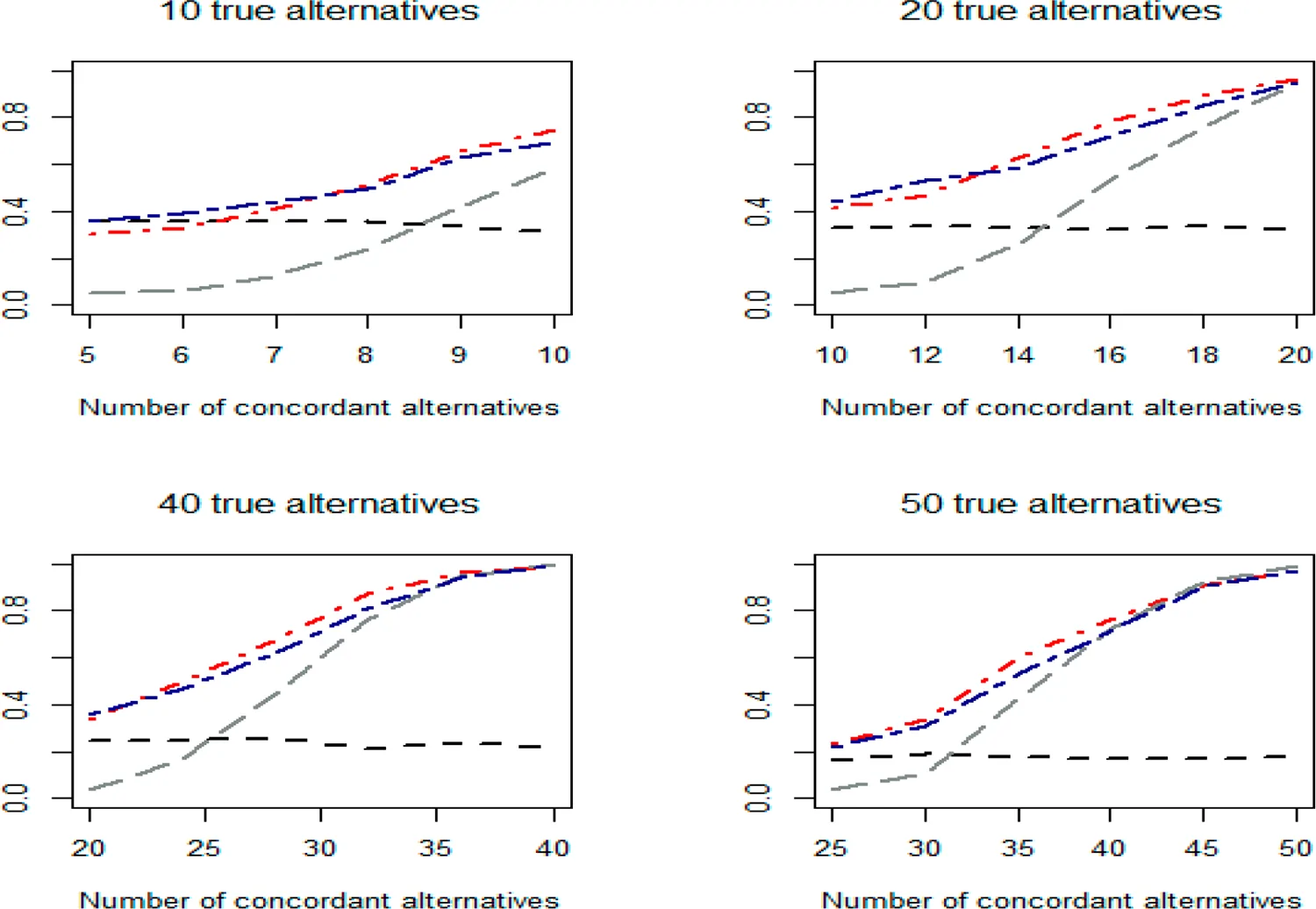
Empirical power values of the tests based on one-sided p-values under Scenario 3.

**Table 1.** Estimated odds ratio and its 95% CI from each study in a meta-analysis with 12 trials. Data were taken from Bachmann et al. [15] and Riley et al. [16].

Study	OR	95% CI	Study	OR	95% CI	Study	OR	95% CI
1	1.11	0.51, 2.39	5	0.88	0.39, 1.95	9	1.06	0.63, 1.79
2	0.97	0.78, 1.21	6	1.28	0.71, 2.30	10	2.95	1.54, 5.63
3	1.13	0.73, 1.72	7	1.19	0.69, 2.08	11	2.36	1.18, 4.72
4	1.08	0.42, 2.75	8	3.82	1.37, 10.60	12	1.68	1.05, 2.70

## Data Availability

The raw data supporting the conclusions of this article will be made available by the authors on request.
